# Williams-Beuren Syndrome and Congenital Lobar Emphysema: Uncommon Association with Common Pathology?

**DOI:** 10.1155/2017/3480980

**Published:** 2017-05-24

**Authors:** Timothy Andrew Walsh, Krishna Revanna Gopagondanahalli, Atul Malhotra

**Affiliations:** ^1^Monash Newborn, Monash Children's Hospital, Melbourne, VIC, Australia; ^2^Department of Paediatrics, Monash University, Melbourne, VIC, Australia

## Abstract

**Introduction:**

Congenital lobar emphysema (CLE) and Williams-Beuren Syndrome are two rare conditions that have only been reported together in a single case study.

**Case Presentation:**

We report another case of a male Caucasian newborn with nonspecific initial respiratory distress, with detection of CLE on repeat chest X-ray on Day 25 of life and concurrent ventricular septal defect, supravalvular aortic stenosis, and branch pulmonary stenosis, in whom a 7q11.23 deletion consistent with Williams-Beuren Syndrome was made.

**Conclusion:**

A diagnosis of congenital lobar emphysema should prompt further screening for congenital heart disease and genetic deletion, and further research is needed to investigate the role of elastin gene mutation in the development of the neonatal lung.

## 1. Introduction

Williams-Beuren Syndrome (also known as Williams syndrome) is a rare contiguous gene deletion disorder with an estimated prevalence of 1 : 7500 live births with typical facial features, cardiovascular anomalies, growth failure, skeletal abnormalities, hypercalcemia, and a distinct neurodevelopmental and behavioral profile [1].

Congenital lobar emphysema (CLE) is a rare respiratory disorder characterized by overdistension of a pulmonary lobe presenting as respiratory distress due to compression of the adjacent and opposite healthy lung tissue resulting in ventilation perfusion mismatch. The incidence of CLE is one in 20,000–30,000 with variable clinical and radiographic manifestations [2].

While Williams-Beuren Syndrome is a complex multisystem disorder known to affect various systems, its association with significant respiratory disorders is not well established. We present a case of a male infant born with heart defects and congenital lobar emphysema, later confirmed to have Williams-Beuren Syndrome. To the best of our knowledge there is only one other case previously reported with this unusual association [3].

## 2. Case Presentation

A male baby was born by emergency caesarean section at 32 weeks' gestation due to decreased fetal movements to a 38-year-old Caucasian mother. A single dose of antenatal steroids was given before the delivery. The mother had well-controlled gestational diabetes but was otherwise in good health. There was no family history of significant respiratory disease or cardiac anomalies. Antenatal anomaly scans were normal.

**Figure 1:**
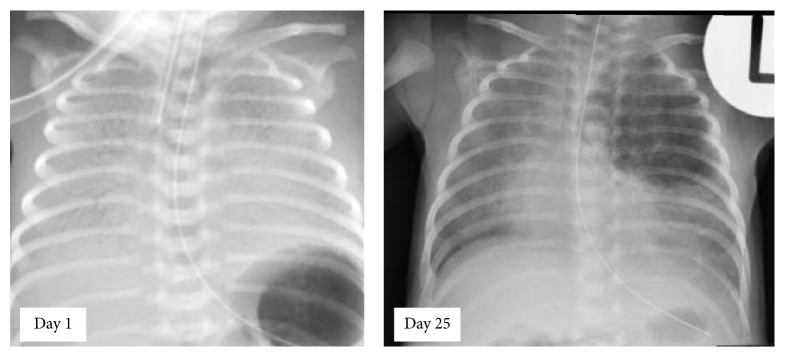
Chest radiographs of neonate showing features of hyaline membrane disease on day 1 of life and typical features of left upper lobe congenital lobar emphysema (CLE) on day 25 of life.

The baby was born with Apgar scores of 7 at 1 and 5 minutes and an Apgar of 9 at 10 minutes, requiring continuous positive airway pressure (CPAP) support for initial respiratory distress (admission blood gas: pH 7.2 pCO_2_ 67, base excess −1). The birth weight was 1464 g (9th percentile), head circumference was 27 cm (2nd percentile), and length was 38 cm (2nd percentile). The baby was intubated at 4 hours of life due to an increasing oxygen requirement (FiO_2_ up to 0.5) and required 2 doses of surfactant therapy. His initial chest X-ray was consistent with hyaline membrane disease (Figure 1), and the patient was subsequently successfully weaned and extubated to nasal CPAP on day 3 of life.

On clinical examination, he was also noted to have a grade 3/6 holosystolic murmur on the left sternal edge. An echocardiogram demonstrated a moderate-large perimembranous ventricular septal defect (VSD) with left to right shunt and a mild supravalvular aortic stenosis with mild-moderate branch pulmonary artery stenosis. There were some facial dysmorphic features (small upturned nose, long philtrum, and a small chin) but the rest of the clinical examination was unremarkable.

As is standard practice at our center with a known congenital heart disease, a microarray (Agilent SurePrint Custom G3 CGH + SNP, 0.20 Mb resolution, Santa Clara, CA) was sent which demonstrated a male molecular karyotype with a heterozygous ~1.41 Mb deletion within the 7q11.23 regions, involving more than 20 genes including* ELN* (elastin). This deletion was consistent with a diagnosis of Williams-Beuren Syndrome.

During the course of hospital stay, the baby developed progressive significant tachypnea. Physical examination revealed occasional basal crackles and reduced air entry in the left upper zone. Chest X-ray (Figure 1) done on day 25 of life showed a well-defined area of hyperlucency in the left upper zone with mediastinal shift consistent with a diagnosis of congenital lobar emphysema. A postcontrast CT scan thorax confirmed overextension of the left upper lobe with mediastinal shift, in keeping with congenital lobar emphysema.

The patient remained stable on CPAP with mild tachypnea and minimal oxygen requirement. The screening abdominal, renal, and head ultrasound scans done in the 5th week of life were normal, as were his serum calcium levels. He subsequently underwent left upper lobectomy at 6 weeks of age for persistent dependence on respiratory support with good postoperative recovery. The histopathology of the lung lesion demonstrated bullae and overextended alveoli and was consistent with the diagnosis of CLE.

At one year of age, the patient was being followed up by the Pediatric Cardiologists besides a general pediatrician and the home enteral nutrition team and had recently undergone a closure of his moderate sized membranous VSD and enlargement of the right sided pulmonary artery with a GORE-TEX® patch. He remained reliant on nasogastric feeds.

## 3. Discussion

Williams-Beuren Syndrome (WBS) was first described by Williams and Beuren independently in 1961 [4, 5]. It is a rare multisystem genetic disorder caused by a submicroscopic deletion of 1.55 Mb to 1.83 Mb on chromosome 7q11.23, in which almost all cases involve the deletion of the* ELN* gene, which codes for elastin [6]. WBS is considered a contiguous gene deletion as it is not exclusively caused by elastin haploinsufficiency, but the deletion involves a region that spans more than 28 genes [6]. Given the size of the deletion in WBS, the phenotypic variations seen are enormous. The common characteristics features are typical facial features classically described as “elfin” facies, cardiac defects, infantile hypercalcemia, musculoskeletal defects, growth failure, mild to moderate intellectual disability, distinct neurobehavioral profile, and many other minor associations which are beyond the scope of this discussion.* ELN* gene haploinsufficiency has been directly linked to certain cardiac defects seen in WBS, namely, supravalvular aortic stenosis [7], but the relationship with connective tissue anomalies and the characteristic facial features are not clearly established. The association of WBS and significant respiratory disorders, particularly congenital lobar emphysema, is not well established and has been reported in only one case so far [3]. Unlike our patient, this case involved a 2-month old infant with confirmed William-Beuren Syndrome and complex congenital heart disease who was incidentally discovered to have an overinflated right middle lobe at the time of initial cardiac surgery, later confirmed to be CLE on multiple serial chest X-rays, with resection at 3 months of age.

Congenital lobar emphysema is a rare cause of severe respiratory distress in neonates, which is characterized by overdistension of a lobe or segment of lobe by a “check valve” mechanism leading to mediastinal shift and progressive respiratory distress. CLE is more common in males and commonly involves the left upper lobe. An abnormal lobar bronchus development, most often a cartilage defect, and several extrinsic causes have been postulated as a causative mechanism in CLE [8], with chance associations with Miller-Dieker Syndrome and with Fanconi anaemia also reported [9, 10].

The mechanical properties of the normal lung are much influenced by elastin and other connective tissues, mainly collagen [11]. The cross linking of collagen and elastin is crucial for structural and elastic property of healthy lung and is catalyzed by lysyl oxidase which is a copper dependent enzyme [11]. Human elastin gene* (ELN)* is affected in 96% of patients with WBS [12] and is vital for the production of the protein tropoelastin. Elastin is then produced through the cross linking of tropoelastin. Many studies have demonstrated that disrupting lung elastin levels results in some form of emphysema or fibrosis when subjected to injury [11]. In a study by Shifren et al. [13], mice models with low levels of elastin demonstrated a congenital emphysema-like condition when exposed to smoke [14]. In another study, the lungs of copper deficient rats developed emphysematous changes through defective cross linking between elastin and collagen due to decreased lysyl oxidase activity [15]. These findings indicate that dysfunctional elastin makes lung prone for emphysematous changes when exposed to injury/stress, with Wan et al. [16] reporting on at least one lifelong nonsmoking adult patient with WBS who had moderate emphysematous changes on CT scan. They went on to perform spirometry on a small subset of young patients with WBS; although they did not demonstrate significant respiratory impairment they postulated that there was an anecdotally higher incidence of respiratory symptoms such as coughing and wheeze, perhaps due to subclinical emphysema and lung disease which may become more apparent at an older age.

Familial cases of CLE have been reported, indicating an autosomal dominance inheritance pattern. A minor alteration in transcription in homeobox genes* Nkx2.1* has been hypothesized as one possible mechanism of familial CLE [15]; however a literature search to relate* Nkx2.1* with gene defects seen in WBS did not reveal any associations. Until now we know only a few gene defects in WBS; the relationship between other genes in the deleted region of chromosome 7 and the signs and symptoms of Williams-Beuren Syndrome is under investigation or unknown.

The reported incidence of associated cardiac defects in infants with CLE is around 20% [17]. The genetic association (defects in* Nkf 2.5* are associated with cardiac defects) and the close temporal relationship during embryonic development between lung and heart may indicate underlying molecular genetic defect in coexistence of CLE and cardiac defects [18].

## 4. Conclusions

WBS is a rare, complex, multisystem genetic disorder. The occurrence of significant respiratory diseases like congenital lobar emphysema is not a recognized association of WBS. The two recent cases of WBS with CLE including this case may change the understanding of spectrum of clinical features of WBS, and we would recommend screening all cases of CLE for cardiac defects and genetic deletions. We conclude it is either* ELN *gene defects or common gene defects associated with cardiac anomalies that may be the best possible explanation for this rare association, but that further research is needed to delineate the role of elastin in the development of the neonatal lung.
